# Nitrofurantoin: properties and potential in treatment of urinary tract infection: a narrative review

**DOI:** 10.3389/fcimb.2023.1148603

**Published:** 2023-07-27

**Authors:** Marzie Mahdizade Ari, Shirin Dashtbin, Fatemeh Ghasemi, Soheila Shahroodian, Parisa kiani, Elnaz Bafandeh, Talieh Darbandi, Roya Ghanavati, Atieh Darbandi

**Affiliations:** ^1^ Department of Microbiology, School of Medicine, Iran University of Medical Sciences, Tehran, Iran; ^2^ Department of Pathobiology, Division of Microbiology, School of Public Health, Tehran University of Medical Sciences, Tehran, Iran; ^3^ Department of Bacteriology, Faculty of Medical Sciences, Tarbiat Modares University, Tehran, Iran; ^4^ Department of Medical Biotechnology, Faculty of Medicine, Lorestan University of Medical Sciences, Khorramabad, Iran; ^5^ Department of Pharmacy, Tehran Medical Sciences, Islamic Azad University, Tehran, Iran; ^6^ School of Medicine, Behbahan Faculty of Medical Sciences, Behbahan, Iran; ^7^ Molecular Microbiology Research Center, Shahed University, Tehran, Iran

**Keywords:** Nitrofurantoin, urinary tract infection, drug-resistant uropathogen, fluoroquinolones, side effects

## Abstract

Nitrofurantoin (NF), a wide-spectrum antibiotic accessible since 1953, is utilized widely to treat urinary tract infections as it usually stays active against drug-resistant uropathogen. The use of Nitrofurantoin has increased exponentially since new guidelines have repositioned it as first-line therapy for uncomplicated lower urinary tract infection (UTI). To, although fluoroquinolones are usually used to re-evaluate the first- and second-line therapies for treating uncomplicated UTI, their level of utilization is thought to be inappropriately excessive and will eventually have a detrimental impact; thus, we hypothesize that NF might be the best choice for this condition, because of its low frequency of utilization and its high susceptibility in common UTI pathogens. It can be concluded from this review that NF can be considered as the most effective drug in the treatment of acute urinary infection, but due to the long-term side effects of this drug, especially in elderly patients, it is essential to introduce some criteria for prescribing NF in cases of chronic UTI.

## 1 Introduction

Nitrofurantoin (NF) was identified in 1953 and was first recommended for the treatment of cystitis in 2010 according to the Infectious Diseases Society of America (IDSA) guideline (Sanchez et al., 2016).

Currently, 150 million Urinary Tract Infections (UTIs) are reported annually worldwide, and drug-resistant infections usually require more complex treatment regimens and are more likely to occur if treatment fails (Khoshbayan et al., 2022). NF is outstanding because NF is most extensively utilized in humans, and its effectiveness in the treatment of lower UTIs and prophylaxis is well established (Conklin, 1978). It is presently prescribed as first-line UTI medical care due to the emergence of resistance to different antibiotics such as carbapenem resistance (Conklin, 1978; Garau, 2008; Gupta et al., 2011; Matthews et al., 2016; Mohebi et al., 2016; Kazemian et al., 2019). It has bacteriostatic and bactericidal effects and is instantly excreted in high concentrations by the kidneys (Komp Lindgren et al., 2015; Fransen et al., 2016). NF is bacteriostatic in low concentrations (5-10 pg/mL) and bactericidal in higher concentrations (Andriole, 1985). Other studies refer to the therapeutic or prophylactic use of this antibiotic. In therapeutic application, 50–100 mg q6h (regular-release formulation) or 100 mg q12h or q8h (slow-release formulation) and in prophylaxis, 50–100 mg q24h is recommended (McOsker and Fitzpatrick, 1994; Cunha et al., 2017; Fransen et al., 2017). With this background, we summarized the NF data available as a valuable choice in the treatment of acute urinary infection, but due to the long-term side effects of this drug, especially in elderly patients, it is essential to introduce some criteria for prescribing NF in cases of chronic UTI.

## 2 Pharmacology (Pharmacokinetic and Pharmacodynamics) and Biochemistry of NF

**Figure 1:**
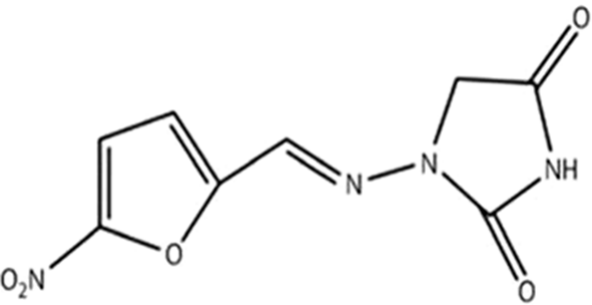
Chemical structure of NF (Wijma et al., 2018).

NF is a redox-active antibacterial agent with the molecular formula of C8H6N4O5 and the molecular weight of 238.16, and is an oral antibiotic based on nitrofurans (Dos Santos et al., 2021). NF, which is a member of the nitrofuran family composed of a furan ring [five-membered aromatic ring with four carbon (C) atoms and one oxygen (O)] is directly linked to a nitro group (-NO2) (FDA. Macrodantin^®^ (NF Macrocrystals) Capsules Product Information. Cincinnati, OH, USA: Procter & Gamble Pharmaceuticals, 2009; 1–12.) (**Figure 1**). In the market, NF is available in oral forms of capsules, tablets, and suspension (oral suspension also known as furadantin). NF is often prescribed in a dose of 50-100 mg 4 times a day for 5 days (Gardiner et al., 2019; Dos Santos et al., 2021). **Table 1** summarizes these characteristics of NF. In terms of PK, NF quickly reaches its therapeutic concentration level, so 90% of NF is quickly exerted through the urine and that is why all its therapeutic effects are restricted to the treatment of UTI. NF is 80-90% orally bioavailable and its bioavailability is about 38.8-44.3%. It has a short half-life (20 minutes), is active only in the urinary tract, and has no other systemic activity. Also, it is rapidly absorbed and eliminated, with low plasma protein binding to plasma proteins or tissues. It is well absorbed from the gastrointestinal tract and excreted unchanged in urine (25–40%) and bile. It has been reported that NF can accumulate in urine, with its effect enhanced by the acidic pH of urine. The anti-bacterial activity of NF and its metabolites is improved under acid conditions. Metabolites are formed by bacterial enzyme reduction, but the precise structure and antibacterial activity of each metabolite remains uncertain (Beckett and Robinson, 1958).

**Table 1:** Characteristics of NF.

Antibiotic	NF
Formula	C8H6N4O5
Molecular weight	238.16
Bioavailability	38.8-44.3%.
PK	90% of NF quickly exerted through the urine,
Pharmacodynamics	Disruption of Krebs cycle and interference in metabolism of carbohydrates, biosynthesis of proteins, cell wall and DNA
Dosage	50-100 mg 4 times a day for 5 days
Clinical use	Urinary tract infection
Adverse effects	Renal, pulmonary, hepatic and nerve failure/Drug induced fever

## 3 Effect of food on PK

Most absorption of NF is done in the duodenum, so the presence of food in GI tract leads to an increase in the time of gastric emptying. Therefore, more NF dissolves in gastric juice before it reaches the duodenum (Jaffe and JM, 1975).

The dissolution time hypothesis is supported by the results of Naggar and Khalil, who showed that absorption increased when the solubility of NF was increased by the addition of Mg2O8Si3 (Naggar and Khalil, 1979).

## 4 Impact of NF on UTI

**Figure 2:**
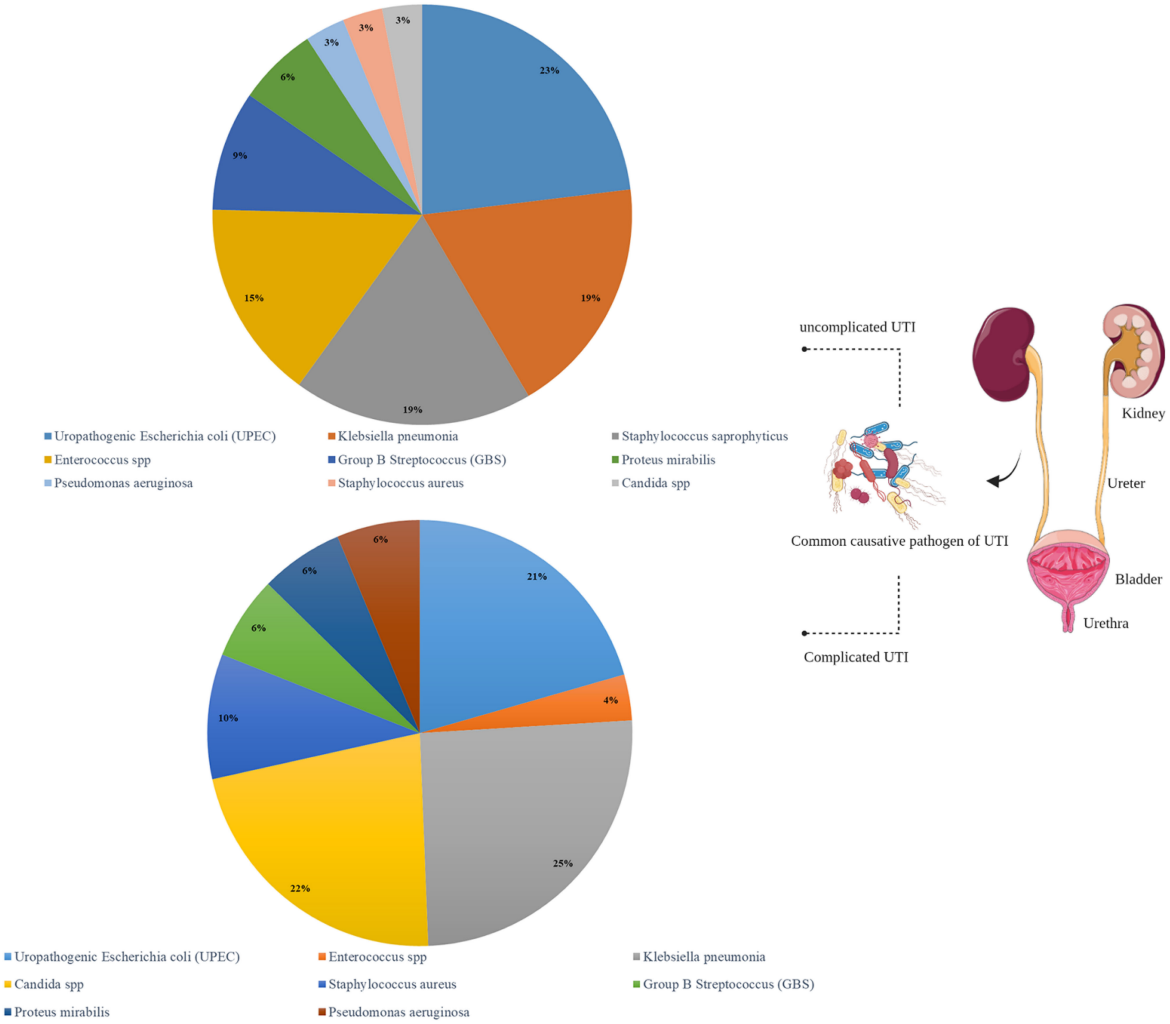
Complicated and uncomplicated UTI prevalence as well as causative pathogens.

UTI is one of the most common bacterial infections and has two complicated and uncomplicated forms which are differentiated by symptoms and causative agents (**Figure 2**).

The uncomplicated form of UTI is often caused by uropathogenic Escherichia coli (UPEC) strains in 80% of cases (Klein and Hultgren, 2020), followed by Klebsiella pneumoniae, Staphylococcus saprophyticus, Enterococcus spp., and group B Streptococcus (GBS). Dysuria, frequency and urgency, suprapubic pain and hematuria are the most common symptoms of UTI (Chew et al., 2019; Ghanavati et al., 2018a). This infection occurs mostly in all aged women (50-60), boys and the elderly. Moreover, the predisposing factors include age, level of sexual activity of people and pre-existing underlying disease (Klein and Hultgren, 2020). Untreated UTI cases can cause sepsis with or without pyelonephritis (Flores-Mireles et al., 2015), leading to death in 10-30%; therefore, UTI patients may sometimes need to be admitted to the hospital (Komagamine et al., 2022). Values less than or equal to 15 µg/mL are suitable for eliminating E. coli (common cause of UTI) and more than 100 µg/mL for eliminating Enterobacter spp. and Klebsiella spp (Cunha, 1988).

Conventional antibiotic therapy for acute uncomplicated UTI includes trimethoprim-sulfamethoxazole, Cefpodoxime, Cephalexin and Cefuroxime, Ciprofloxacin, Cefepime, Ampicillin, Imipenem/Cilastatin and Trimethoprim-Sulfamethoxazole are suitable choices for acute complicated form (Long and Koyfman, 2018). The emergence of antibiotic-resistant strains and elimination of the microbial flora of the gastrointestinal tract and vagina may occur following long-term use of these conventional antibiotics in patients suffering from UTI (Kostakioti et al., 2012; Flores-Mireles et al., 2015). Fosfomycin and NF are two alternative antibiotics to prescribe in cases of resistant strains, but NF is more effective than fosfomycin and shows a greater effect on pregnant women (Gardiner et al., 2019; Ghanavati et al., 2018b). Oral prescription of NF in both liquid (25 mg/5 ml) and solid (100 mg) forms shows an optimum effect on the treatment of UTI. Studies have shown that oral NF is the best choice for prophylaxis before surgery and the treatment of patients over age 12. Therefore, NF is currently used prophylactically in UTI cases specially against vancomycin-sensitive and resistant strains (VRE and VRS) associated with catheters as well as fluoroquinolones and aminopenicillins resistant strains (Cunha, 2006). This can be the only antibiotic that is effective in treating enterococcal strains instead of ampicillin.

Twenty-seven trials consisting of 4807 patients have been conducted to analyze NF as a remedy for UTIs. NF was determined to be clinically and microbiologically effective, with clinical cure rates between 79% to 92% and microbiological eradication rates of 80%–92%.

## 5 Antimicrobial effect of NF

In addition to the greatest effect on uropathogens, NF has an inhibitory effect on a wide range of Gram-positive bacteria such as Staphylococcus and Enterococcus and Gram-negative bacteria such as Klebsiella and Citrobacter (Munoz-Davila, 2014). It seems that E. coli as the most bacteria isolated from the uncomplicated UTI cases is more inactivated by NF among other Gram-negatives, while Enterobacter, Klebsiella, Citrobacter and Providencia are less effective, and Pseudomonas, Proteus, Acinetobacter, Morganella and Serratia are completely ineffective and show resistance to NF (Naber et al., 2008; Gardiner et al., 2019). Mouse urinary tract infection models have shown that the MIC required for treatment with NF in an animal model is much lower than antibiotics such as Sulfamethoxazole/Trimethoprim, Fosfomycin, Mecillinam, Ciprofloxacin, and Cefdinir, and will eliminate more live bacteria (Nakagawa et al., 2021).

## 6 Mechanism of action and resistance rates

**Figure 3:**
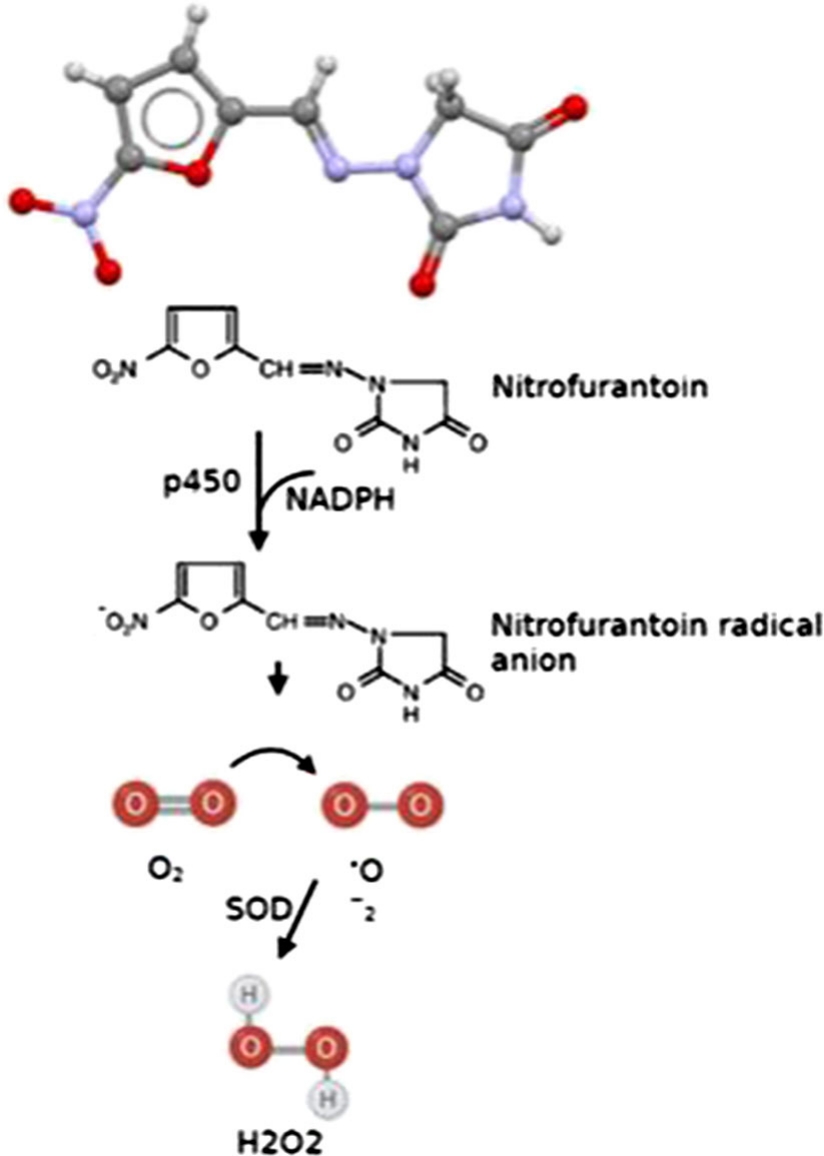
Scheme of NF mode of action and ROS generation. p450, cytochrome P450 reductase; NADPH, Nicotinamide Adenine Dinucleotide Phosphate Hydrogen; SOD, superoxide dismutase; H2O2, hydrogen peroxide.

Following the activation of the nitro group in its molecular structure by the cytochrome P450 reductase (**Figure 3**), NF affects the protein synthesis machinery and ribosome in susceptible bacteria (Wang et al., 2008) and disrupts Krebs cycle (citric acid cycle) by inhibiting a series of enzymes involved in the metabolism of carbohydrates (Cunha, 1989; Munoz-Davila, 2014), as well as cell wall and DNA. This interference with vital processes leads to bacterial death (Munoz-Davila, 2014).

For the first time, the resistance to nitrofurantoin has been reported in *E. coli* which attributed to the presence of a mutation in the gene coding for nitrofurantoin (*nfsA*), an oxygen-insensitive enzyme. This mutation prevents the reduction of NF and the subsequent production of toxic compounds (Race et al., 2005). In addition to chromosomal gene, plasmid-mediated NF-resistant strains also shows higher MIC and target *nfsA* and *nfsB* genes (Sastry and Jayaraman, 1984; Ho et al., 2016). While the NF breakpoint is defining as 32 mg/L, some resistant strains in anaerobic conditions, shows decrease in the MIC value. This is explained by the activation of the oxygen system and the presence of oxygen-sensitive nitroreductase (type II), which are activated in the absence of oxygen-insensitive type I reductases. Gautam et al. in study which conducted in 2021, showed the increase in multi drug resistant strains while there is no effective drug, has led to an increase in the prescription of NF and its increasing resistance rate (Gautam et al., 2021). In response to whether NF-resistant isolates will emerge or not, it should be mentioned that although the frequency of mutations in resistance to NF is high, treatment failure seems to be rare, and considering that most urinary tract infections are treated empirically, the desired antibiotic first must be determined based on the sensitivity pattern therefore the importance is to investigate NF-resistant strains to see whether they are still treatable or not (Sandegren et al., 2008).

Bacterial flavoproteins can reduce the drug, leading to reactive electrophilic intermediates that change or inactivate bacterial molecules (Gleckman et al., 1979; McOsker and Fitzpatrick, 1994). As a prodrug, NF is activated by two kinds of oxygen-insensitive nitroreductases, nfsA and nfsB (Le and Rakonjac, 2021). High levels of resistance to NF (median MIC of 96 μg/ml) are principally mediated by mutations in nfsA and/or nfsB (encoding oxygen-insensitive nitroreductases) (Shakti and Veeraraghavan, 2015). Deletions in ribE also result in resistance due to inhibition synthesis of Riboflavin/Flavin (vital cofactor of NfsA and NfsB( (Vervoort et al., 2014; Sekyere and Asante, 2018). Efflux pumps are the other factors that play a role in resistance to this antibiotic (Ho et al., 2016). A study reported resistance rates of nitrofurantoin from 2011 to 2019. In E. coli, Klebsiella spp, Proteus spp. and Enterococcus spp. resistance rates were 4.8%, 46.0%, 100.0% and 4.8%, respectively (Hrbacek et al., 2020). The low resistance rate in E. coli and Extended Spectrum Beta-Lactamase (ESBL) producing Enterobacteriaceae may be due to different mechanisms of action (Huttner et al., 2015).

A study conducted by Ahmed et al. showed that the pattern of antibiotic resistance in E. coli as the most common pathogen causing UTI, was as follows: Ampicillin (86%), Amoxicillin (76%), Tetracycline (71%), Trimethoprim-Sulfamethoxazole (64%), Cephalexin (61%) and Cephalothin (60%), respectively. Also, this strain has the highest antibiotic sensitivity to Imipenem (86%), NF (82%), Amikacin (79%) and Ciprofloxacin (72%) (Ahmed et al., 2019). According to a systematic review by Bryce et al., the prevalence of antibiotic resistance to such common antibiotics in UTIs caused by E. coli such as Ampicillin, Trimethoprim, Co-amoxiclav, Ciprofloxacin and NF largely differ in different countries. In this way, the OECD (Organization for Economic Co-operation and Development) countries have much less antibiotic resistance, which is attributed to the availability of common antibiotics. In other words, NF in some countries have much lower antibiotic resistance than in non-OECD countries. When common antibiotics are routinely used in the treatment of UTIs, they have contributed more to antibiotic resistance (Bryce et al., 2016). Regarding the effect of NF on resistant pathogens, Tulara et al. evaluated the effect of Fosfomycin and NF on extended-spectrum-beta-lactamase-producing E. coli (ESBL-EC), and the results indicated the effectiveness of NF in the ESBL-EC (Tulara, 2018). Moreover, FQ-resistant E. coli, are not only affected by NF, but this antibiotic has also provided a cost-effective feature (McKinnell et al., 2011).

## 7 Adverse effects of NF

Antibiotic resistance is considered as one of the possible side effects of any antimicrobial agents. This issue has been reported in long-term prophylaxis cases for UTI and elderly patients with renal failure. Generally, NF is considered as a safe antimicrobial drug, but, in 1 per 100,000 patients (Vickery et al., 2022) in long-term use, there may be some risks. The non-drug resistance side effects of NF like hepatotoxicity, neuropathy and pulmonary damages are directly related to the long-term use of this drug (Wang et al., 2008). Three complications, Gastrointestinal (GI) and skin manifestations and peripheral neuropathy (Tan et al., 2012), are the most important and serious adverse effects of NF consumption, respectively. The first warning about the risks of NF in the elderly was given in 2003 (Fick et al., 2003), and then in 2012 NF was listed among the potentially dangerous drugs causing renal failure in elderly patients (Fick et al., 2012). It is important to mention that despite the serious reactions (e.g. renal, pulmonary, hepatic failure and nerve adverse effects) in elderly patients, the occurrence of these AEs is very rare (Rego et al., 2016). Pulmonary syndrome can also appear in the first hours to the first week of drug prescription which is accompanied by fever, chills, and cough (Munoz-Davila, 2014). This serious and lethal reaction to nitrofurantoin was diagnosed on the first day of admission by Kanji et al. which was eventually treated by intubation and corticosteroids (Kanji et al., 2011). In this case report, the patient had received Trimethoprim 100 mg for cystitis which was replaced by NF 100 mg prescription due to lack of any progress. Following another course of NF, an 82-year-old man (with no history of underlying disease or smoking) presented to the hospital with symptoms of dyspnea, fever, and cough (Kanji et al., 2011). The incidence rate of such complications is less than 1% following frequent and long-term use of NF (for 6 months to years), and mostly in women younger than 60 years old (Guidance et al., 2002; Vahid and Wildemore, 2006; Fenton et al., 2008). According to the American Geriatrics Society Beers Criteria Update of 2012, using NF for a long time in elderly patients with renal failure must be banned. Moreover, some researchers prefer nitrofurantoin to be considered as a second treatment choice not a primary agent (Fick et al., 2012).

According to *in vitro* studies, long-term consumption of NF damages DNA by inhibiting DNA synthetase and chromosomal mutations. Moreover, Tumorigenicity of NF has not yet been precisely proven, but there is no doubt that NF has the ability to produce toxic metabolites (Lawson et al., 2016). Further studies are needed in this field.

### 7.1 Liver injury due to NF

Drug-induced liver injury (DILI) can result in an acute or chronic hepatitis-like syndrome. The acute form is usually associated with 1 to 2 weeks of NF treatment and is rare (approximately 0.3 per 100,000 prescriptions). Acute liver injury usually occurs within weeks of starting treatment with NF and may occur within weeks of completing a defined course of treatment. The more common form of hepatotoxicity is due to chronic prophylactic use, occurring in 1 in 1,500 people (National Institute of Diabetes and Digestive and Kidney Diseases (US), 2012). A wide range of hepatotoxicity has been reported in association with NF use, including acute hepatitis, granulomatous reaction, cholestasis, or autoimmune hepatitis to chronic active hepatitis that can lead to cirrhosis or death. The mechanism is not fully understood, but is thought to be due to an immunological reaction or a direct cytotoxic response. It has been hypothesized that prolonged therapy to NF, female gender, older age, and impaired renal function increase the risk of hepatotoxicity. Corticosteroids have been used in conjunction with stopping NF to treat severe cases (Sakaan et al., 2014). Genetic predisposition including HLA-B8 appears to increase the risk of NF-induced liver injury (Burgert et al., 1995; Stine and Northup, 2016). The correlation between the dose and NF-induced liver injury is contradictory. Lower and higher doses of NF are effective in long-term prophylaxis (Muller et al., 2017). Prophylactic choice in recurrent cystitis is controversial, but must be based on evaluating the patient, risks and benefits as well as hepatotoxicity (Byron, 2019). Although there are no guidelines, monitoring liver enzymes at 1 to 3 months interval during therapy is recommended (Sherigar et al., 2012). **Table 2** mentions the cases suffering from liver disorders due to the use of this antibiotic.

**Table 2:** Patients with liver disorders due to the use of this antibiotic.

Author	Age/G	Medication	Dosage	Pattern	Latency	Recovery	Other Drug histories	Ref
Luk T et al.	53y, F	Nitrofurantoin	100 mg twice daily	Portal-hepatic vasculature, hepatic nodularity, prominent parenchymal necrosis and collapse and accompanying cholestatic hepatic encephalopathy and ascites	12 M after restarting	Death	LOR	(Luk et al., 2021)
Wonnacott S et al.	24y, F	Nitrofurantoin	50 mg every six hours	Epigastric pain	Past 3 D	After stopping	Not Reported	(Wonnacott et al., 2022)
Appleyard S et al.	65y, F	Nitrofurantoin	50 mg daily	Chronic inflammatory cell infiltrate with interface hepatitis and piecemeal necrosis in portal areas	6 Y	After only one M of prednisolone	Mb, dosulepin, LAN, PCT, inhaled SALB, and intermittent Fcz	(Appleyard et al., 2010)
	42y, F	Nitrofurantoin	50 mg daily	Marked chronic inflammation within the portal tracts and extensive fibrosis, and some features of cirrhosis	2 Y	After a few weeks of corticosteroid medication	FolA	
	74y, F	Nitrofurantoin	100 mg daily	Striking lobular inflammation, confluent and bridging necrosis, syncytial giant cells, minimal portal inflammation and minimal plasma cells	2 Y	Seven months after initial presentation	Not Reported	
Khan F et al.	56y, F	Nitrofurantoin	Not reported	Abdominal pain	14 D	Over the course of 3 D	Not Reported	(Khan et al., 2019)
Koulaouzidis et al.	57y, F	Nitrofurantoin	100 mg at night	Hepatitis	16 M	At 4 M follow up	SALB inhaler, be-clathasone inhaler, AM, and LAN	(Koulaouzidis et al., 2007)
Hydes T et al.	50y, F	Nitrofurantoin	50 mg once daily	Biliary obstruction autoimmune chronic active hepatitis with mild fibrosis, in keeping with immune-mediated drug-induced liver injury	12 M prior to admission	Two months later	PIO, Met, AM, PAX, LAN, CPM, BUP, MSO4, temazepam and LOS	(Hydes et al., 2014)
	75y, F	Nitrofurantoin	50 mg once daily	Chronic active hepatitis with a florid inflammatory cell infiltrates consistent with primary or drug-induced AIH on a background of cirrhosis	6 M later	One month postadmission, the patient developed a pulmonary embolus	PRO, ramipril, BFTZ and AT	
Carvalho de Matos A et al.	68y, F	Nitrofurantoin	100 mg once daily	Portal tract moderate mononuclear cell inflammatory infiltrate, with mild plasmacytes and some eosinophils, severe interface hepatitis with focal emperipolesis, periportal hepatocellular rosetting and ballooning, and ductular reaction. Severe panlobular bilirubinostasis, focal lobular necroinflammatory activity, and mild to moderate portal fibrosis (Masson trichrome) were also observed as well as focal periportal copper deposit (rhodamine).	2 Y	Not Reported	AX, clavulanic acid, and Cla	(Carvalho de Matos et al., 2022)

D, day; M, month; Y, year; NR, not reported; LOR, lorazepam; Mb, Mebeverine; LAN, Lansoprazole; PCT, Paracetamol; SALB, salbutamol; Fcz, Fluconazole; FolA, folic acid; PIO, pioglitazone; Met, Metformin; AM, amitriptyline; PAX, paroxetine; CPM, Chlorphenamine; BUP, Buprenorphine; MSO4, morphine sulfate; LOS, lactulose; PRO, Propranolol; BFTZ, Bendroflumethiazide; AT, atorvastatin; AX, amoxicillin; Cla, clarithromycin; AM, amitriptyline.

### 7.2 Fever following the consumption of nitrofurantoin

Drug-induced fevers are independent-infection conditions which are classified as the miscellaneous group and may induce fever which is known as fever of unknown origin (FUO). FUO is characterized as temperatures higher than 38.3°C which will elapse more than two weeks after they appear (Haidar and Singh, 2022). According to estimations, 4-7% of empirical antibiotic therapy performed in hospitalized patients induce FUO (Patel and Gallagher, 2010; Vickery et al., 2022). NF in oral non-suspension form may be associated with FUO (Roth and Basello, 2003). However, the occurrence of these fevers is directly related to patients with an impaired immune system, especially neutropenic patients (Patel and Gallagher, 2010), so it is important to discuss it. A case report published in 2022 observed clozapine-induced fever (CIF) in a 60-year-old woman who was receiving medication related to schizoaffective disorder (Vickery et al., 2022). Fever was reported in this patient following antibiotic therapy with three doses of NF. The fever did not follow a regular pattern. Also, laboratory analyses have shown an increase in eosinophil and lactate dehydrogenase. Apart from the 39.3 C fever, use of nitrofurantoin by the patient indicated the occurrence of bradycardia. Similar to these results, another cohort study indicated an increase in the incidence of fever and allergy in the group receiving nitrofurantoin compared to the sulfonamide group (Koch-Weser et al., 1971). Forster et al. attributed the occurrence of such an adverse reaction to the frequent and repeated use of NF in their case report (Forster et al., 2009).

## 8 The response spectrum of patients with UTI to the prescription of nitrofurantoin

As shown in **Table 3**, NF is well-tolerated by patients with UTI following both prophylaxis and treatment (Fisher et al., 2018). NF is often given as prophylaxis for 3 days. Cohort studies have indicated the beneficial effect of nitrofurantoin on patients, like a study by Huttner et al. who reported the cure rate of nitrofurantoin at 70% (Huttner et al., 2018). NF is safe and effective for short-term treatment at younger ages which is in contrast with the low effect of nitrofurantoin in patients who use nitrofurantoin for the treatment of UTI for a longer period of time (Gardiner et al., 2019). Antibiotic treatment in symptomatic patients (i.e., UTI caused by uropathogens) seems more successful than in asymptomatic patients, probably due to the effective targeting of pathogens by antibiotics. However, in asymptomatic patients only uropathogens are colonized (do not show any activity), and the treatment is less successful. Having a series of virulence factors in bacteria which make them a target, tolerance in bacteria in the presence of antibiotics, and inaccessible areas to antibiotics activity (e.g., presence of uropathogens in the bladder) are the most important reasons for the ineffectiveness of antibiotics in preventing the occurrence of UTI (Fisher et al., 2018). Today, possible adverse effects of prophylaxis of nitrofurantoin in the emergence of antibiotic-resistant strains have become a concern as we will discuss in the next section (Goff and Mendelson, 2017).

## 9 Nitrofurantoin-resistant bacteria in patients with UTI

As shown in **Table 2**, improvement of symptoms in symptomatic UTI patients, incidence rate of UTI in prophylaxis use of nitrofurantoin and occurrence of antibiotic resistance are important outcomes in assessing the effects of nitrofurantoin. Generally, resistance to nitrofurantoin is less common even in drug-resistant strains (Sanchez et al., 2016). However, a few strains of E. coli isolated from urine and K. pneumoniae producing ESBL enzyme (Procop et al., 2003) have been reported to show resistance to nitrofurantoin. The prevalence of NF resistant in E. coli strains isolated from UTI cases in the United States and France has been reported at 1.1% and 1.8%, respectively (Zhanel et al., 2005; Honderlick et al., 2006). However, 99% of E. coli, 69% of Klebsiella strains and 63% of Enterobacter strains are still sensitive to nitrofurantoin, while the resistance level of conventional drugs used in UTI against E.coli such as ciprofloxacin and trimethoprim/sulfamethoxazole is reported at 25-29% (Vs 2.3%) which is more than nitrofurantoin’s resistance rate (Mazzulli et al., 2001; Kashanian et al., 2008). On the other hand, antibiotics such as Fluoroquinolones and Cotrimoxazole, which were conventionally prescribed for the treatment of uncomplicated UTI, today seems to have lost their effectiveness due to the emergence of antibiotic resistance, so nitrofurantoin and fosfomycin are suitable alternatives due to less drug-resistant cases (Munoz-Davila, 2014). In addition to the clinical use of nitrofurantoin, antibiotic prophylaxis before surgery outcomes for prevention of UTI is shown in **Table 3**. Clinical trials have shown that the use of nitrofurantoin in the group that used this antibiotic as prophylaxis, compared to the control group which used conventional drugs for UTI such as trimethoprim and co-trimoxazole, led to more antibiotic resistance. This was the most important result of the study by Fisher et al. in which patients had received nitrofurantoin for 9-12 months before surgery (Fisher et al., 2018). Moreover, these findings are consistent with a study by Pickard et al. who found that bacteria isolated from patients who had taken oral nitrofurantoin prophylactically showed more antibiotic resistance (Pickard et al., 2018). Researchers pointed to complications caused by the long-term use of nitrofurantoin; therefore, the use of this drug as a preventive tool was avoided and fluoroquinolones were prescribed instead which led to an increase in fluoroquinolone-resistant strains (Slekovec et al., 2014). Therefore, it is advisable the use of nitrofurantoin be limited to the treatment of UTI cases which show resistance to other antibiotics.

**Table 3:** Clinical efficacy of Nitrofurantoin on UTI by investigation randomized clinical trials (RCTs).

Author	Year	Sex	Age (years)	Clinical use (prophylaxis or treatment)	Route of nitrofurantoin administration	Dosage/Duration	Outcomes (recovery/bacterial colony count) If prophylaxis, do experience UTI or not?	Adverse effects (AEs)	Cure rate/Incidence rate (IR) of UTI	Ref
**Bastawros et al.**	2021	F	61.6 ± 11.7	PEP	Capsule	5 D/100 mg twice- OD	Did not reduce the risk of UTI	NS/GI manif (nausea)	IR= 18%	(Bastawros et al., 2021)
**Akinci et al.**	2021	46/59 (F: M)	4.8 ± 3.9	PEP	Capsule	Single dose of 1 mg/kg	Significant reduction in risk of UTI	NG	3.8% reduction	(Akinci et al., 2021)
**Lavelle et al.**	2020	F	61.7 ± 61.9	PEP	Capsule	100 mg OD	Did not reduce the risk of UTI	AEs were common/RES to NF were found	IR= 17.3%	(Lavelle et al., 2019)
**Fisher et al.**	2018	115/88 (F: M)	59.1	PEP	Capsule	50 mg once OD	Significant reduction in risk of UTI	NS/ GI, skin and fungal manif. RES to NF were found commonly	IR=1·3 cases per person; 0·52	(Fisher et al., 2018)
**Pickard et al.**	2018	115/88 (F: M)	X≥18	PEP	Capsule	50 mg once OD	Significant reduction in risk of UTI	RES to NF were found commonly	IR=0·52	(Pickard et al., 2018)
**Huttner et al.**	2018	F	X≥18	Treatment	Tablet	5 D/100 mg 3 times OD	Improvement in symptoms/less than 10^3^ CFU/ml	NS/ GI manif	72%	(Huttner et al., 2018)
**Gupta et al.**	2007	F	18-45	Treatment	Tablet	5 D/100 mg twice OD	Improvement in symptoms/less than 10^5^ CFU/ml	NS/ GI, UG and NEUR manif	88%	(Gupta et al., 2007)
**Christiaens et al.**	2002	F	15-55	Treatment	Capsule	3 D/100 mg four times OD	Improvement in symptoms/less than 10^5^ CFU/ml	NS/ GI, skin, UG and NEUR manif	81%	(Christiaens et al., 2002)
**Stein**	1999	F	X≥12	Treatment	Capsule	7 D/100 mg	Improvement in symptoms/less than 10^5^ CFU/ml	NS/ GI and UG manif	69.5%	(Stein, 1999)
**Iravani et al.**	1999	F	X≥18	Treatment	Capsule	7 D/100 mg	Improvement in symptoms/less than 10^3^ CFU/ml	NS/ GI and UG manif	83%	(Iravani et al., 1999)
**Hooton et al.**	1995	F	X≥18	Treatment	Capsule	3 D/100 mg four times OD	less than 10^2^ CFU/ml	NS/ GI, UG and NEUR manif	61%	(Hooton et al., 1995)

PEP, Antibiotic post-exposure prophylaxis before surgery; D, day; OD, Once a day; NS, Not serious; NG, Not given; UG, Urogenital; NEURm Neurological; manif, manifestations; RES, Resistance.

## 10 Combined effects of nitrofurantoin with different antimicrobial agents

Extensive experimental use of antibiotics to treat various infectious diseases has increased antibacterial resistance among many strains of pathogenic bacteria worldwide (Ayaz et al., 2019). Combining antibiotic treatments with other treatments is becoming an increasingly important strategy for treating many of these infections, especially those caused by pathogens with antibiotic resistance (Fatsis-Kavalopoulos et al., 2020). One of the advantageous features of combination therapies is the synergism effect. The therapeutic effect is greater when an antibiotic is combined *in vitro* than the sum of each drug (Coates et al., 2020). The synthetic antibiotic NF is used to treat lower urinary tract infections orally (Dos Santos et al., 2021). By reviewing the studies conducted so far, which are summarized in **Table 4**, we found that the combination of NF and other antibiotics has not been extensively studied. Moreover, most of the studies conducted in this field have been done in laboratory conditions. The results of these studies show that in most cases, the effect of NF increases in combination with other antibiotics (except in combination with mecillinam) (Fatsis-Kavalopoulos et al., 2020). It seems that NF antimicrobial combination therapy is superior to monotherapy, but using drug combinations has many challenges, including simultaneous assessment of distribution and tissue penetration, among others (Zhong et al., 2020).

**Table 4:** Results of the combinations of nitrofurantoin with other Antibiotics.

Authors/References	Year	Study	Country	Pathogen	Source of pathogen	Combination antibiotics	Effects (synergistic, antagonistic, additive or no effect)
R Daza et al. (Daza et al., 1977)	1997	Mic St	Spain	Gram-Negative bacilli	Pathological products of hospital	NF +FOS	No effect
JL Descourouez et al. (Descourouez et al., 2013)	2013	Mic St	USA	VRE	USI	NF +FOS	No effect
Nikos Fatsis Kavalopouos et al. (Fatsis-Kavalopoulos et al., 2020)	2020	*CombiANT methodology	Sweden	E. coli	UTI	NF +CIP	Additive
						NF +TMP	Synergistic
						NF +MEC	Antagonistic
Alice Zhou et al. (Zhou et al., 2015)	2015	Mic St	USA	Escherichia coli mutants**	–	NF +VAN	Synergistic
Peng Cui et al. (Cui et al., 2016)	2016	Mic St and Ani St	China	E. coli Persisters	UTI	NF +COL	No effect *in vitro*, Additive *in vivo*
Abdulkareem H.ABD et al. (ABD et al., 2014)	2014	Mic St	Iraq	E. coli	UTI	NF +CN	Synergistic
						NF +CIP	No effect
Zi-Xing Zhong et al. (Zhong et al., 2020)	2020	Mic St and Ani St	China	MDR UPEC	UTI	NF +AK	Synergistic

Nitrofurantoin (NF), Fosfomycin (FOS), Ciprofloxacin (CIP), Gentamycin (CN), Vancomycin (VAN), amikacin (AK), colistin (COL), mecillinam (MEC), ciprofloxacin (CIP), trimethoprim (TMP), Microbiological Study (Mic St), animal study (Ani St), Vancomycin-Resistant Enterococcus faecium (VRE), Urinary Tract Infection (UTI), Urinary Stent Infections (USI), Uropathogenic E. coli (UPEC).

*CombiANT methodology: a 3D-printed agar plate insert that produces defined diffusion landscapes of 3 antibiotics, permitting synergy quantification between all 3 antibiotic pairs with a single test.

** mutant E. coli strains (dcd and surA mutants) that have increased sensitivity to VAN.

Another noteworthy point is that in recent years, researchers have done considerable research on the effect of various bioactive compounds in combination with antibiotics. The scientific and medical community has been exploring the possibility of creating synergistic therapeutic regimens by combining plant extracts and nanoparticles [especially silver nanoparticles (AgNPs)]. There is growing evidence that the use of these substances enhances the antibacterial properties of conventional antibiotics, repurposing them instead of replacing them (Cheesman et al., 2017; Vazquez-Muñoz et al., 2019). Combinations of natural compounds may make it possible for antimicrobial agents to interact better with their targets within pathogens and prevent resistance. Such a strategy can reduce toxicity, because lower concentrations of both agents can be used in this method (Betoni et al., 2006; Sanhueza et al., 2017). Furthermore, because nanoparticles are so small, they stick to the cell wall in addition to damaging it. For this reason, NPs are less resistant to antibiotics than antibiotics (Betoni et al., 2006). The antimicrobial action of NPs is also influenced by metal ions and reactive oxygen species (Khleifat et al., 2022). So far, many studies have been conducted on the combined effect of NF with these substances, and we mentioned a few of them (**Table 5**). The results of these studies showed that the combination of NF with nanomaterials or plant extracts has increased the effectiveness of this antibiotic. But since in other studies (Moussaoui and Alaoui, 2016; Paralikar et al., 2019), some materials in combination with antibiotics had an antagonistic effect, it is necessary to conduct more studies on the combination of substances with this antibiotic. Also, more studies on the mechanism of the antagonistic effect of these substances are necessary.

**Table 5:** The effect of nitrofurantoin combination with some plant extracts and nanoparticles.

Authors/References	Year	Country	Pathogen	Combined treatment		Effects (synergistic, antagonistic, additive or no effect)
				Plant extract	Nanoparticles	
**Ngalah Bidii Stephen et al.** **(** Frank et al., 2022)	2022	Germany	Serratia marcescens	NF + Iso^1^		Synergistic
**Shatha Mousa mlaghee Al-safi et al.** **(** Al-safi et al., 2020)	2022	Iraq	salmonella	NF + Phoenix dactylifera^2^		Synergistic
**Ali M Khlaifat** **et al.** (Khlaifat et al., 2019)	2022	Jordan	Pseudomonas aeruginosa		NF + AgNPs	Synergistic
			Pseudomonas aeruginosa	NF + C. Sempervirens and A.graveolens^3^		Synergistic
			E. aerogenes	NF + C. Sempervirens and A.graveolens^3^		Synergistic
			S. aureus	NF + A.graveolens^3^	NF + AgNPs	Additive
**Rajendran Mala et al.** **(** Mala et al., 2017)	2017	India	Escherichia coli		NF + SNPs	Synergistic

^1^ Isothiocyanates: Natural plant products generated by enzymatic hydrolysis of glycosylates; ^2^ Oily extraction of leaves; ^3^ Essential oils; Silver nanoparticles (AgNPs), Silver nanoparticles (SNPs).

Generally, understanding the mechanism of action of antibiotics, AgNPs, plants and combined treatments allows predicting more feasible treatments or designing new ones more efficiently. Even if some aspects of the mechanism of action remain unknown, these results provide a more effective way to fight infectious diseases (Vazquez-Muñoz et al., 2019).

## 11 Conclusion

In this review, our goal was to obtain a comprehensive picture by considering the clinical use of nitrofurantoin and its adverse effects to inform physicians to manage UTI patients under long-term nitrofurantoin therapy. Nitrofurantoin should not be recommended for long-term prophylaxis in patients with UTI, especially elderly patients. The intention is not to discard nitrofurantoin prescription, but urologists must use nitrofurantoin as the most effective drug on acute UTI. Therefore, a series of supervisions and criteria regarding the prescription of nitrofurantoin in cases of chronic UTI are needed.

## Author contributions

AD and RG conceived, designed and supervised the study. AD and MM contributed to data collection, interpretation and final approval of data for the work. SD and FG developed the first and final draft of the manuscript. SS and PK developed the second draft of the manuscript. All figures and tables were designed and checked by MM, EB and TD. All authors reviewed and contributed to the revisions and finalized the drafts.
